# Evaluating components of dental care utilization among adults with diabetes and matched controls via hurdle models

**DOI:** 10.1186/1472-6831-12-20

**Published:** 2012-07-09

**Authors:** Monica Chaudhari, Rebecca Hubbard, Robert J Reid, Ronald Inge, Katherine M Newton, Leslie Spangler, William E Barlow

**Affiliations:** 1 Washington Dental Service, 9706 Fourth Avenue NE, Seattle, WA, 98115, USA; 2 Group Health Research Institute, 1730 Minor Ave, Seattle, WA, 98101, USA; 3 Dept of Biostatistics, University of Washington, Seattle, WA, 98105, USA; 4 Cancer Research and Biostatistics, 1730 Minor Ave, Seattle, WA, 98101, USA

## Abstract

**Background:**

About one-third of adults with diabetes have severe oral complications. However, limited previous research has investigated dental care utilization associated with diabetes. This project had two purposes: to develop a methodology to estimate dental care utilization using claims data and to use this methodology to compare utilization of dental care between adults with and without diabetes.

**Methods:**

Data included secondary enrollment and demographic data from Washington Dental Service (WDS) and Group Health Cooperative (GH), clinical data from GH, and dental-utilization data from WDS claims during 2002–2006. Dental and medical records from WDS and GH were linked for enrolees continuously and dually insured during the study. We employed hurdle models in a quasi-experimental setting to assess differences between adults with and without diabetes in 5-year cumulative utilization of dental services. Propensity score matching adjusted for differences in baseline covariates between the two groups.

**Results:**

We found that adults with diabetes had lower odds of visiting a dentist (OR **=** 0.74, p **<** 0.001). Among those with a dental visit, diabetes patients had lower odds of receiving prophylaxes (OR **=** 0.77), fillings (OR **=** 0.80) and crowns (OR **=** 0.84) (p** < **0.005 for all) and higher odds of receiving periodontal maintenance (OR **=** 1.24), non-surgical periodontal procedures (OR **=** 1.30), extractions (OR **=** 1.38) and removable prosthetics (OR **=** 1.36) (p < 0.001 for all).

**Conclusions:**

Patients with diabetes are less likely to use dental services. Those who do are less likely to use preventive care and more likely to receive periodontal care and tooth-extractions. Future research should address the possible effectiveness of additional prevention in reducing subsequent severe oral disease in patients with diabetes.

## Background

Diabetes mellitus (DM) is a chronic metabolic disorder characterized by a deficiency in insulin secretion or an increased insulin resistance, resulting in hyperglycemia. Oral complications including gingivitis, periodontitis, oral soft-tissue pathologies and tooth loss are common in people with diabetes [1-5], with an estimated prevalence of about 1/3 (Newton et al. 2011) [6]. While the clinical pathology of oral diseases associated with diabetes has been extensively studied, there is limited research on diabetes associated dental care utilization. Although adults with diabetes experience an increased oral disease burden, this may not translate into increased utilization due to differences in care seeking patterns. Studying dental care utilization among adults with and without diabetes may reveal significant behavioral differences or need for new approaches to bridging gaps in disease management.

The contributions of this paper are two-fold. First, we developed a new econometric model for dental services utilization that closely represents the data distribution and allows for differential effects of risk factors on different facets of the utilization distribution (i.e. the use of dental services and the type and amount of care received). This gives each part an attractive structural interpretation and facilitates targeting of tailored interventions to address the parts of the utilization distribution most impacted by risk factors. Second, we used this model to investigate differences in use of dental care services between adults with and without diabetes.

In modeling the usage of dental services, we propose to adapt hurdle or two-part models that have served as a methodological cornerstone of empirical analysis in the health economics literature [7]. The hurdle model has been popular, partially because it describes the dual decision structure of the utilization process. The first component of this process consists of the decision to make contact with the health care system and is influenced largely by individual patient-level factors. The second component of the process consists of the specific course of treatment undertaken and can be influenced by physician as well as patient-level factors. In other words, the factors impacting the decision to seek dental care may differ from those that affect the type or amount of care received. We propose a variation of the hurdle model in which dental utilization is decomposed into: (1) a component consisting of the decision to seek treatment, (2) a component describing the specific type of treatment received by the patient, and (3) a component describing the amount or intensity of treatment received by the patient. Separate likelihood-based estimation is then proposed for each component of the model.

In this study, we present the above described hurdle model for dental utilization and apply this technique to examine dental care utilization among adults with and without diabetes from a large population-based cohort. In this cohort we investigated the probability of seeking dental care, as well as the likelihood and intensity of types of dental services received, including prophylaxes (cleanings), periodontal procedures (maintenance, surgical and non-surgical), restorations (fillings, crowns), extractions, and prosthetics.

## Methods

### Data sources & study period

This study was conducted using linked data from Washington Dental Service (WDS) and Group Health Cooperative (GH). As described elsewhere [8], medical and dental records from these two sources were linked with a 96% success rate on matching variables. WDS is a founding member of the Delta Dental Plans Association delivering dental care to more than two million people through employer-sponsored programs. GH is a nonprofit health care system that coordinates care and coverage to more than half a million residents of Washington state and Idaho. Secondary enrollment data from WDS and GH, clinical (laboratory, pharmacy and diagnosis) data from GH, and claims data from WDS were obtained for enrollees continuously and dually insured between January 1, 2002 and December 31, 2006. We examined each participant’s utilization of dental services for the 5-year study period using WDS claims data. All study procedures were approved by Group Health Cooperative’s Institutional Review Board.

### Inclusion criteria, exposure groups, dental outcomes and baseline covariates

Dually insured enrollees meeting all of the following criteria were selected as study participants: 1) continuous insurance enrollment, allowing for coverage interruptions caused by employment changes of no longer than 90 days; 2) age 40–70 years as of 1/1/2002; 3) enrollment in GH’s integrated health care delivery system; 4) at least one clinical encounter with a medical provider using utilization data during 2002–2006 to assess important confounding variables like smoking status and BMI; 5) no diagnosis of organ transplantation, HIV/AIDS, dementia, or cancer (except non-melanoma skin cancers) occurring during the study period; 6) enrollment in indemnity and PPO dental benefit plans, that use similar fee-for-service methods for provider reimbursement.

**Figure 1:**
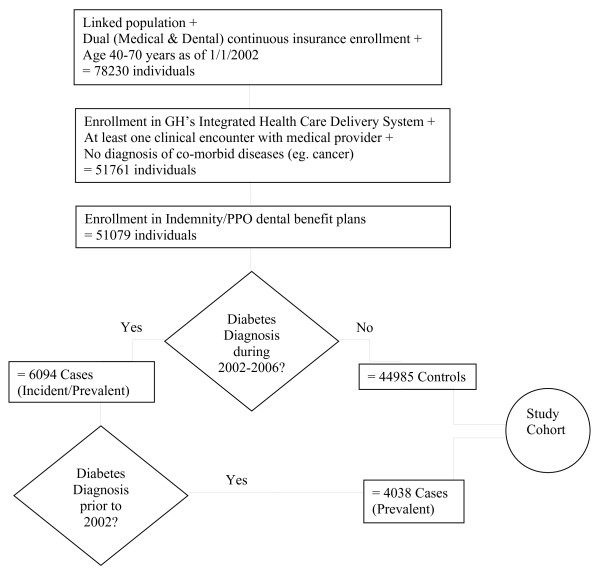
Flow-chart for study cohort.

Using GH’s clinical data, participants were identified with a diagnosis of diabetes if any of the following criteria were met: two fasting plasma glucose measurements ≥ 126 mg/dl, or two non-fasting glucose measurements ≥ 200 mg/dl, or one of each within a 12 month period; glycosilated hemoglobin ≥ 7.0%; filled prescriptions for insulin or oral hypoglycemic agents; hospitalizations with a primary or secondary diagnosis of diabetes (250.xx). This definition of a diabetes case is consistent with the definition validated in prior research [9]. To be included in the study’s diabetes cohort, we required that the diabetes-diagnosis criterion was met at least once in 2002–2004 and also at least once in the period 36 months prior to this date. The purpose of these two requirements was to ensure that cases had well-established diabetes by 2002 and that they continued to seek treatment during the study period. Participants with an instance of diabetes not satisfying the above two requirements or with diagnoses of gestational diabetes (648.0x, 648.8x) were excluded from the study cohort. Figure 1 describes the number of patients meeting each inclusion/exclusion criterion. 

Our outcome of interest was cumulative dental utilization obtained from dental claims submitted during the 5 year follow-up period. We categorized utilization based on procedure classifications from the American Dental Association Coding system - Current Dental Terminology (CDT) which were further refined to include more specific codes (See Additional file 1: Appendix D). High concordance between the database and actual patient records on selected procedures has been demonstrated in prior validation research [10].

Baseline covariates of interest were selected a priori on the basis of theoretical relevance and were grouped as demographics, health-behaviors, baseline co-morbidities and access to health care (Table 1). Demographics included age groups (40–45, 46–50, 51–54, 55–59 and 60–70) and sex. Smoking status and body mass index (BMI) that were routinely collected at health care encounters were extracted from GH’s electronic medical records and were used as proxies for health-behaviors. Recognizing that smoking is a strong risk factor for periodontal disease, an individual’s status was defined as non-smoking if at least 90% of the medical encounters between 2002 and 2006 were recorded as non-smoking, to allow for some clinical recording errors. BMI was grouped into five categories (≤ 18.5, 18.6-24.9, 25–29.9, 30–39.9, and 40+) ranging from underweight to morbidly obese based on the median of recorded values within a 3-year window centered at 2002. To account for differences in preventive health care seeking behavior, we also controlled for the use of medical well care visits (none, one, or more than one) during the five year study period. Because baseline co-morbidity may confound the effect of diabetes on prospective dental utilization, we used a standardized, non-proprietary predicted chronic disease co-morbidity score for each subject for 2002 based on their age, sex, insurance status and types of outpatient pharmacy dispenses in the previous year [11-13]. In statistical models, we used the log transformed co-morbidity score to account for the skewed distribution of this predictor. As use of ACE-inhibitors and statins, also representing co-morbidity, is linked to dental outcomes like tooth loss [14], we grouped subjects into four categories based on their usage of either, both or none of these two drugs in 2002. Since access to health care could vary between regions and type of medical insurance, using WDS claims data, we split the regions of care delivery in Washington state into metropolitan King county, regions east, west, north and south relative to King County, and outside the state which we defined as ‘other’. Medical insurance plans obtained from the GH enrollment databases were classified as Medicare, Government, and Individual/Commercial/other. 

**Table 1:** Baseline characteristics by diabetes status and dental visit status

	**Diabetes status (exposure)**				**Dental visit status (outcome)**	
	**Controls**		**Adults with diabetes**		**% with no dental visit**	**No. of services given at least 1**
	**(n = 44985)**		**(n = 4038)**		**(N = 49023)**	**visit**
	**No.**	%^*^	**No.**	%^*^	**%**^**↑**^ **= 10%**	**Mean ( SD)**
**Age**						
40-45	9,768	22	399	10	9	26.0 (12.4)
46-50	11,203	25	708	18	9	27.2 (13.0)
51-54	8,991	20	809	20	9	28.2 (13.4)
55-59	7,503	17	933	23	10	29.0 (13.9)
60-70	7,520	17	1,189	29	12	29.4 (14.1)
**Sex**						
Female	24,113	54	1,824	45	8	27.9 (12.9)
Male	20,872	46	2,214	55	11	27.8 (13.9)
**Smoking Status**						
No	36,958	82	3,439	85	8	28.1 (12.9)
Yes	6,960	15	590	15	17	26.6 (15.5)
**BMI**						
Underweight-Normal	11,684	26	339	8	6	28.5 (12.5)
Overweight	15,664	35	979	24	8	28.3 (13.2)
Obese	8,605	19	1,114	28	11	27.7 (13.8)
Clinically Obese	3,521	8	718	18	12	27.4 (14.2)
Morbidly Obese	2,125	5	682	17	14	26.1 (14.1)
**Well Care Visits**						
0	11,771	26	1,726	43	17	25.9 (14.4)
1	13,720	31	1,212	30	10	27.4 (13.6)
> = 2	19,494	43	1,100	27	5	29.2 (12.4)
(5.30 - 6.48)	14837	33	241	6	9	26.6 (12.5)
(6.48 - 6.80)	5136	11	91	2	8	27.9 (12.8)
(6.80 - 7.37)	8790	20	279	7	9	27.9 (13.0)
(7.37 - 7.95)	9258	21	492	12	9	28.5 (13.4)
(7.95 - 11.17)	6857	15	2,922	72	12	28.6 (14.9)
**Ace-Statin Usage**						
No Ace-Statin	39,804	88	1,142	28	9	27.8 (13.0)
Only Ace	2,615	6	1,691	42	14	27.5 (13.6)
Only Statin	1,628	4	208	5	10	28.8 (13.8)
Both Ace-Statin	938	2	997	25	14	27.7 (14.9)
**Region**						
King County	19,666	44	1,511	37	6	28.9 (13.4)
North	4,809	11	428	11	7	28.5 (13.3)
East	232	1	27	1	15	27.4 (14.1)
South	6,001	13	664	16	12	27.1 (14.2)
West	13,822	31	1,351	33	11	26.4 (12.8)
**Insurance Group**						
Medicare	3,560	8	566	14	12	29.1 (13.9)
Ind-Com-Other	14,264	32	996	25	6	28.9 (13.1)
Government	27,161	60	2,476	61	11	27.1 (13.4)
**Diabetes Status**						
No	-		-		9	27.9 (13.2)
Yes	-		-		16	26.9 (14.9)

Missingness on Smoking Status (2.2%), BMI (7.3%), Co-morbidity (0.2%); Out of state (1.04%).

* Column Percentages; ^↑^ Row Percentages.

### Hurdle model for dental utilization

A variation of the hurdle or two-part model, which can be used to model sequential stages of utilization [15-18] was used to analyze the association between diabetes and dental utilization. In this model, we decompose the likelihood function describing dental utilization into three separate components, specified by parametrically independent likelihood functions, which allows for separate estimation of each stage of utilization. This model allows us to separately examine factors that may influence (1) use of any dental services, (2) the specific type of dental procedures received among those receiving any services, and (3) the quantity or intensity of a specific dental procedure received among those receiving each type of service.

Let *V* be a binary variable which assumes a value *v*_*i*_ = 1 if an individual *i* visited a provider with probability *P*_*i*_, and 0 otherwise. Let *U*_*t*_ be a binary variable which assumes a value *u*_*it*_ = 1 if an individual *i* received a procedure in treatment class *t* with probability (1 - *π*_*it*_) and 0 otherwise. Let *y*_*it*_*, y*_*it*_ > 0, with probability mass function *f*_*t*_*(y*_*it*_*)*, represent the number of times an individual *i* received a procedure in treatment class *t*. Then the likelihood for the three components can be expressed as

(1) $$\begin{array}{l} P\left(V=v_{i}\right)=\{\begin{array}{c} p_{i}\text{;}\;v_{i}=1\\ 1-p_{i}\text{;}\;v_{i}=0 \end{array}\\ P\left(U_{t}=u_{\mathrm{it}}|V=1\right)=\{\begin{array}{c} 1-\pi_{\mathrm{it}}\text{;}\;u_{\mathrm{it}}=1\\ \pi_{\mathrm{it}}\text{;}\;u_{\mathrm{it}}=0 \end{array}\\ P\left(Y_{t}=y_{\mathrm{it}}|V=1,U_{t}=1\right)=f_{t}\left(y_{\mathrm{it}}\right)/\left(1-f_{t}\left(0\right)\right);y_{\mathrm{it}}>0 \end{array}$$

The marginal distribution of *Y*_*t*_ given a dental visit and unconditional distribution of *Y*_*t*_ are then given by

(2) $$\begin{array}{c} P\left(Y_{t}=y_{\mathrm{it}}|V=1\right)=\{\begin{array}{c} \;\pi_{\mathrm{it}}\text{;}\;y_{\mathrm{it}}=0\\ \left(1-\pi_{\mathrm{it}}\right)f_{t}\left(y_{\mathrm{it}}|y_{\mathrm{it}}>0\right)\text{;}\;y_{\mathrm{it}}>0 \end{array}\\ P\left(Y_{t}=y_{\mathrm{it}}\right)=\{\begin{array}{c} \left(1-p\right)\;v_{i}=0,y_{\mathrm{it}}=0\\ \pi_{\mathrm{it}}p_{i}\text{;}\;v_{i}=1,y_{\mathrm{it}}=0\\ p\left(1-\pi_{\mathrm{it}}\right)f_{t}\left(y_{\mathrm{it}}|y_{\mathrm{it}}>0\right)\text{;}\;v_{i}=1,y_{\mathrm{it}}>0 \end{array} \end{array}\;$$

and the log-likelihood function is

(3) $$\begin{array}{c} LogLik=\left\{\sum_{v_{i}=0,y_{\mathrm{it}}=0}1n\left(1-p_{i}\right)+\sum_{v_{i}=1,y_{\mathrm{it}}=0}1np_{i}+\sum_{v_{i}=1,y_{\mathrm{it}}>0}1np_{i}\right\}\\ +\left\{\sum_{v_{i}=1,y_{\mathrm{it}}=0}1n\pi_{\mathrm{it}}+\sum_{v_{i}=1,y_{\mathrm{it}}>0}1n\left(1-\pi_{\mathrm{it}}\right)\right\}\\ +\left\{\sum_{v_{i}=1,y_{\mathrm{it}}>0}1n\left[f_{t}\left(y_{\mathrm{it}}\right)/\left(1-f_{t}\left(0\right)\right)\right]\right\} \end{array}$$

where the first two terms can be regarded as log-likelihood functions for the binary outcomes representing use of any dental services and use of dental services in a specific treatment class, respectively, and the third as a log-likelihood function for a truncated-at-zero count representing the number of services used within a specific treatment class. In this study, we parameterized the first two components using two logit models, such that

(4) $$\begin{array}{l} p_{i}|\gamma =\frac{\exp\left(X_{i}^{'}\gamma \right)}{\left\{1+\exp\left(X_{i}^{'}\gamma \right)\right\}}\\ \&\\ \pi_{\mathrm{it}}|\theta =\frac{1}{\left\{1+\exp\left(X_{i}^{'}\theta \right)\right\}} \end{array}$$

and the third by a zero truncated negative binomial model (ZTNB), such that

(5) $$f\left(y_{\mathrm{it}}|\mu ,\alpha ,X_{i},y_{\mathrm{it}}>0\right)=\left\{\frac{\Gamma \left(\alpha^{-1}+y_{\mathrm{it}}\right)}{\Gamma \left(\alpha^{-1}\right)\Gamma \left(y_{\mathrm{it}}+1\right)}\right\}\times {\left\{\frac{1}{\left({\left(1+\alpha \mu_{i}\right)}^{\frac{1}{\alpha }}-1\right)}\right\}}^{-\alpha^{-1}}\times {\left\{\frac{\mu_{i}}{\left(\mu_{i}+\alpha^{-1}\right)}\right\}}^{y_{\mathrm{it}}}$$

where α represents the over-dispersion term of the negative binomial distribution with $\mu_{i}=\exp\left(X_{i}^{'}\beta \right)>0$. Thus, under two conditions, first, when *v*_*i*_ = 1 and second, when y_it_ > 0, the expected values of the resulting conditional distributions are respectively given by

(6) $$\begin{array}{l} E\left[y_{\mathrm{it}}|\mu ,\alpha ,V=1,\pi \right]=\frac{\left(1-\pi \right)\mu_{i}}{1-{\left(1+\alpha \mu_{i}\right)}^{-\frac{1}{\alpha }}}\\ \&\\ E\left[y_{\mathrm{it}}|\mu ,\alpha ,y_{\mathrm{it}}>0\right]=\frac{\mu_{i}}{1-{\left(1+\alpha \mu_{i}\right)}^{-\frac{1}{\alpha }}} \end{array}$$

and the relative risk of conditional mean utilization given a change in binary explanatory variable are respectively given by

(7) $$\begin{array}{l} \frac{E\left[y_{\mathrm{it}}|\mu ,\alpha ,V=1,\pi ,x_{i}=1\right]}{E\left[y_{\mathrm{it}}|\mu ,\alpha ,V=1,\pi ,x_{i}=0\right]}\\ =\exp\left\{\begin{array}{l} \log\left(E\left[y_{\mathrm{it}}|\mu ,\alpha ,V=1,\pi ,x_{i}=1\right]\right)\\ -\log\left(E\left[y_{\mathrm{it}}|\mu ,\alpha ,V=1,\pi ,x_{i}=0\right]\right) \end{array}\right\}\\ =\exp\left(\theta_{1}\right)*\exp(\beta_{1})*\left\{\frac{1+\exp\left(X\theta \right)}{1+\exp\left(\theta_{1}\right)\exp\left(X\theta \right)}\right\}*\left\{\frac{1-{\left(1+\alpha \exp\left(X\beta \right)\right)}^{-\frac{1}{\alpha }}}{1-{\left(1+\alpha \exp\left(\beta_{1}\right)\exp\left(X\beta \right)\right)}^{-\frac{1}{\alpha }}}\right\} \end{array}$$

&

(8) $$\begin{array}{l} \frac{E\left[y_{\mathrm{it}}|\mu ,\alpha ,y_{\mathrm{it}}>0,x_{i}=1\right]}{E\left[y_{\mathrm{it}}|\mu ,\alpha ,y_{\mathrm{it}}>0,x_{i}=0\right]}\\ =\exp\left\{\log\left(E\left[y_{\mathrm{it}}|\mu ,\alpha ,y_{\mathrm{it}}>0,x_{i}=1\right]\right)-\log\left(E\left[y_{\mathrm{it}}|\mu ,\alpha ,y_{\mathrm{it}}>0,x_{i}=0\right]\right)\right\}\\ =\exp(\beta_{1})*\left[\frac{1-{\left(1+\alpha \exp\left(X\beta \right)\right)}^{-\frac{1}{\alpha }}}{1-{\left(1+\alpha \exp\left(\beta_{1}\right)\exp\left(X\beta \right)\right)}^{-\frac{1}{\alpha }}}\right] \end{array}$$

where $\theta_{1}$ &$\beta_{1}$are the estimated coefficients of a binary predictor in the logit and ZTNB components of hurdle model regression.

However, alternative specifications could be used to model the above components. We fitted the hurdle model to each class of dental utilization to investigate differences between adults with diabetes and controls for each treatment class and each component of utilization.

To facilitate the interpretation of results comparing adults with diabetes to those without, we present odds ratios, estimated by the two logistic components and relative risk ratios of conditional means (given vi = 1 and then yit > 0) estimated by the negative binomial components truncated at zero. The odds ratio from the first stage logistic component estimates relative odds of a dental visit. The second stage conditional relative risk estimate (equation 1 above) gives the multiplicative increase in the average number of procedures received in a specific class given that the participant had a dental visit. We additionally estimate the relative odds of receiving a procedure in a treatment class and relative intensity of such treatment conditional on receiving a treatment in that class. This odds ratio estimates the relative odds of receiving a procedure within a specific class given that the participant had at least one dental visit. And, the relative intensity estimate from the third component (equation 2 above) is interpreted as the multiplicative increase in the average number of procedures received in a specific class given that the participants had at least one procedure in that class.

### Control of confounding

The standard means of isolating independent effects of exposures in observational or quasi-experimental research is to control for observable differences between exposure groups using regression methods. However, regression modeling of confounders requires that we assume a known functional form for the relationship between confounders and the outcome. Additionally, if the distribution of confounders differs substantially between exposure groups, referred to as sparse overlap on observed covariates, then regression-based adjustment for confounding may perform poorly [19]. In our analyses of dental utilization among adults with and without diabetes we therefore chose to use propensity score matching [20-22] to control for confounding.

We constructed propensity scores using a logistic regression model. This model estimated the probability of a diabetes diagnosis given a set of baseline characteristics. After constructing our propensity model, we used PSMATCH2 (version 3.1.5) [23] to match controls to participants with diabetes using a nonparametric kernel matching estimator. We used a tricube kernel and explored two bandwidths, 0.008 and 0.03. While traditional matching algorithms use only a few observations from the comparison group, kernel matching uses weighted averages of all individuals in the control group to construct a counterfactual population of controls matched to the study population of patients with diabetes. By using more information from control individuals, this approach provides greater efficiency. Larger weights are awarded to controls with propensity scores closely matched to that of cases. This approach assigns cases a weight of 1 and closely matched controls’ weights summing to the number of cases. Post-matching standardized differences (SD^†^) in covariates across the two groups were assessed and compared with baseline standardized differences to insure that no significant differences between the populations persisted after matching. Likelihood ratio tests were conducted to assess improvement in overall covariate balance.

After adjusting for confounding via matching, we explored empirical differences between the diabetes and control populations by using matching weights to compute average exposure effects (AEE – see Additional file 1: Appendix A) [24,25]. We then compared groups with and without diabetes on 1) the proportion that used any dental service, 2) the average number of procedures received among those with at least one dental visit, 3) the proportion using each class of dental service among those with at least one dental visit, and 4) the average number of procedures in each treatment class among those with at least one dental visit. *T-*tests were used to detect significant differences between groups. Sensitivity analyses were conducted to assess the overall impact of the bandwidth selected for the kernel matching procedure [26] and to examine the degree to which unobserved confounding factors could alter AEE results [27-29] (see Additional file 1: Appendices B and C).

The kernel weights derived from the matching estimator were used as probability weights in estimation of the hurdle model. In the model’s regression equations, we included all the covariates that were used in propensity score analysis in addition to other variables that were found to explain variability in dental utilization. We present only the effect of diabetes (the primary predictor) for each outcome. For nonlinear effects (point estimates, significance levels, and confidence intervals), calculations are based on the “delta method”, an approximation appropriate in large samples.

As the total population of cases and controls was weighted in the first stage of the hurdle model, the estimates from this stage were protected from confounding because the covariate distribution was balanced across the two groups. Subsequent stages of the hurdle model condition on making a dental visit and receiving a dental procedure in a specific class, respectively. These models therefore use subsets of the total population with weights within the two groups normalized. Within these subsets, the covariate distribution may differ somewhat between the two populations. However, successful matching at the first stage results in substantial similarities in the covariate distribution at subsequent stages. Exposure effect estimates for these stages are therefore less dependent on the specific post-matching statistical model. The preprocessing strategy of matching on propensity scores equalizes the distributions of exposure groups on observed covariates and is suitable for improving subsequent parametric estimation [30]. A post-matching regression then adjusts for residual variation in the outcome and any small remaining imbalances that may result from conditioning in the second- and third-levels of the model.

## Results

### Study population

Of the 49,023 enrollees who were linked, enrolled in GH’s integrated system and who met the remaining study inclusion criteria, 4,038 (8.24%) were identified as having well-established diabetes.

### Descriptive statistics

Prior to matching study participants by their propensity scores, baseline characteristics were found to be significantly different for the two comparison groups. Adults with diabetes tended to be older, were more likely to be male, were largely insured by Medicare, had higher BMI and co-morbidity scores, were more likely to use ACE inhibitors, and used medical “well care” examinations less frequently (Table 1).

The baseline characteristics were also associated with making a dental visit. Participants with no dental visits were more likely to be older, male, insured by Medicare, and smokers. They had higher BMI and co-morbidity scores and used well care visits less often. Use of dental care services also varied between regions (Table 1).

### Propensity score matching

**Figure 2:**
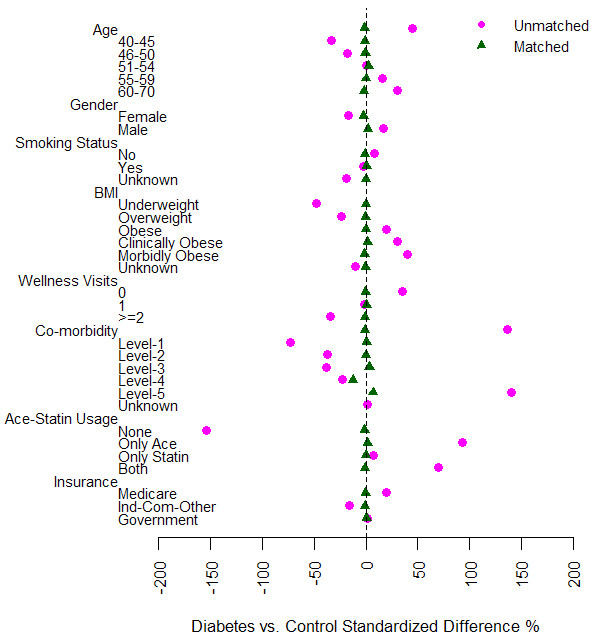
Pre- and post-matching covariate balance.

Our logistic model for diabetes status adequately captured differences between the two groups. The receiver operating characteristic area-under-the-curve was 0.91 indicating fairly accurate classification performance. Controls were matched to the 4038 diabetes patients on propensity scores using the tricube kernel matching estimator with bandwidth 0.008. Eighteen people with diabetes were excluded from subsequent analyses because no control subjects with similar propensity scores could be identified. These subjects were morbidly obese with high co-morbidity scores and used both ACE inhibitors and statins. After matching, no significant differences in baseline covariates persisted. The pseudo R^2^ reduced from 0.37 for the pre-matching model to 0.001 after matching, indicating an overall improvement in covariate balance, and the p-value for the likelihood ratio test for overall model prediction also changed from highly significant (p < 0.001) to non-significant (p = 1.000). Figure 2 demonstrates the improvement in balance achieved between the two groups after matching. All subsequent analyses were limited to this sample with kernel matched weights (N^C^ = 44,985 controls and $N_{bw=0.008}^{D}$ = 4020 diabetics). As a sensitivity analysis, we explored results using a bandwidth of 0.03. Changing the bandwidth did not impact our inference on model results (see Additional file 1: Appendix C). We therefore present only results for bandwidth of 0.008.

### Dental utilization among individuals with and without diabetes

#### Average exposure effect

Compared to controls, individuals with diabetes had a significantly lower probability of visiting a dentist and received fewer dental procedures over the 5-year follow-up period (Table 2). Among those with a dental visit, adults with diabetes when compared with controls had a significantly lower probability of receiving prophylaxes, crowns and fillings and higher probability of receiving periodontal maintenance, non-surgical periodontal procedures (e.g., root planing and scaling), extractions and removable prosthetic procedures. A similar pattern was observed in the average count of procedures received with the exceptions that periodontal maintenance did not differ significantly between the two groups and significantly fewer services in the diagnostic class were received among patients with diabetes compared to controls. Among individuals using any dental services, those with diabetes also used less overall preventive services including both prophylaxes and periodontal maintenance. As expected, the probability of receiving a diagnostic procedure given a dental visit was almost 100%.

**Table 2:** Estimated probability of utilization and average number of procedures used for patients with diabetes and controls following propensity score matching

	**Probability of utilization**				**Average number of procedures**^**↑**^			
	**Diabetes**	**Controls**	**Difference**	**p-value**	**Diabetes**	**Controls**	**Difference**	**p-value**
**Dental Services (overall)**	0.84	0.88	−0.03	< 0.001	22.65	24.54	−1.89	< 0.001
**Services by Dental Class given at least one Dental Visit**								
Diagnostic	0.98	0.98	0.00	0.371	12.04	12.84	−0.81	< 0.001
Prophylaxis	0.72	0.77	−0.05	< 0.001	4.12	4.71	−0.59	< 0.001
Periodontal Maintenance	0.29	0.24	0.04	< 0.001	1.95	1.78	0.17	0.061
Overall Preventive Care^**^**^	0.86	0.89	−0.03	< 0.001	6.07	6.49	−0.42	< 0.001
Periodontal-Non Surgical	0.25	0.20	0.05	< 0.001	0.91	0.72	0.19	< 0.001
Periodontal-Surgical	0.03	0.03	0.00	0.270	0.04	0.05	−0.01	0.128
Filling	0.67	0.72	−0.05	< 0.001	2.79	2.98	−0.19	0.020
Crown	0.49	0.53	−0.04	0.001	1.04	1.17	−0.13	0.001
Extraction	0.33	0.27	0.07	< 0.001	0.96	0.69	0.27	< 0.001
Prosthetic-Fixed	0.11	0.10	0.01	0.063	0.41	0.40	0.01	0.359
Prosthetic-Removable	0.17	0.13	0.04	< 0.001	0.39	0.30	0.09	< 0.001
*Overall					26.91	28.00	−1.09	0.002

^↑^estimates conditional on at least one dental visit but unconditional on at least one service in a specific class. The denominator includes those with no utilization for a given service.

*includes any dental service, not restricted to those mentioned.

^Prophylaxis or Periodontal Maintenance.

#### Sensitivity analysis results

Analyses of sensitivity of our estimates to unobserved confounding indicated that use of any dental services, overall preventive care, extractions and removable prosthetics were robust to confounding (Additional file 1: Appendix B). However, for a few classes of procedures, such as restorations, the existence of unobserved covariates with modest confounding effects could explain the observed association with diabetes.

#### Hurdle model results

Based on the first stage of the model, we found that adults with diabetes, when compared to controls, had 26% lower odds of making a dental visit (OR = 0.74, 95% CI: [0.63, 0.86], p < 0.001) (Table 3).

**Table 3:** Relative odds of dental visit and relative utilization intensity of dental services for patients with diabetes compared to controls based on post-matching hurdle model

	**OR**	**95% CI**		**p-value**
**Dental Visit**	0.74	0.63	0.86	< 0.001
**Relative Utilization Intensity given a Dental Visit**				
	**RR**	**95% CI**		**p-val**
Diagnostic	0.94	0.91	0.97	< 0.001
Prophylaxis	0.80	0.71	0.91	< 0.001
Periodontal Maintenance	1.02	0.93	1.13	0.675
Overall Preventive Care^**^**^	0.82	0.69	0.97	0.018
Periodontal-Non Surgical	1.24	1.07	1.44	0.005
Periodontal-Surgical^**#**^	0.93	0.65	1.33	0.693
Filling	0.97	0.90	1.05	0.482
Crown	0.91	0.83	1.00	0.041
Extraction*	1.23	1.02	1.48	0.031
Prosthetic-Removable	1.24	1.03	1.49	0.021
Prosthetic-Fixed	1.13	0.97	1.31	0.125

^Prophylaxis Or Periodontal Maintenance.

#To avoid collinearity, subjects with missing region, all taking surgical procedures, were randomly assigned to other region groupings.

*Complete convergence was not achieved. Estimates based on last concave results.

We fit the second stage of the model conditional on having made a dental visit. From the relative utilization estimates conditional on making a dental visit (Table 3), we found that among those with a dental visit, diabetes patients received significantly fewer diagnostic procedures (RR = 0.94, 95% CI:[0.91, 0.97]), prophylaxes (RR = 0.80, 95% CI: [0.71, 0.91]), and crowns (RR = 0.91, 95% CI: [0.83, 0.10]) and significantly more non-surgical periodontal procedures (RR = 1.24, 95% CI: [1.07, 1.44]), extractions (RR = 1.23, 95% CI: [1.02, 1.48]) and removable prosthetics (RR = 1.24, 95% CI: [1.03, 1.49], p < 0.001) relative to controls. The group with diabetes had 18% lower utilization of overall preventive care including both prophylaxes and periodontal maintenance.

Upon further exploration of these conditional effects (Table 4), we found that while there was no significant difference in odds of receiving a diagnostic procedure (OR = 0.92, 95% CI: [0.62, 1.36]), among patients receiving diagnostic procedures diabetes patients received 6% fewer (RR = 0.94, 95% CI: [0.91, 0.97]) over the span of 5 years, resulting in overall reduced utilization of procedures in this class. In contrast, diabetes patients had lower odds of receiving prophylaxes (OR = 0.77, 95% CI: [0.68, 0.87]), fillings (OR = 0.80, 95% CI: [0.71, 0.89]) and crowns (OR = 0.84, 95% CI: [0.76, 0.93]) and higher odds of receiving periodontal maintenance (OR = 1.24, 95% CI: [1.10, 1.39]), non-surgical periodontal procedures (OR = 1.30, 95% CI: [1.15, 1.48]), extractions (OR = 1.38, 95% CI: [1.23, 1.54]) and removable prosthetics (OR = 1.36, 95% CI: [1.17, 1.57]) with no significant differences in the utilization intensity in respective classes of treatment given they had such a treatment. For prophylaxes and overall preventive care, diabetes patients had significantly lower intensity of use.

**Table 4:** Relative odds and relative utilization intensity for dental service classes among dental-visit patients with diabetes compared to controls based on post-matching hurdle model

	**OR**	**95% CI**		**p-value**
**Use of Service given a Dental Visit**				
Diagnostic	0.92	0.62	1.36	0.677
Prophylaxis	0.77	0.68	0.87	< 0.001
Periodontal Maintenance	1.24	1.10	1.39	< 0.001
Overall Preventive Care^^^	0.76	0.65	0.90	0.001
Periodontal-Non Surgical	1.30	1.15	1.48	< 0.001
Periodontal-Surgical	0.86	0.62	1.18	0.350
Filling	0.80	0.71	0.89	< 0.001
Crown	0.84	0.76	0.93	0.001
Extraction	1.38	1.23	1.54	< 0.001
Prosthetic-Removable	1.36	1.17	1.57	< 0.001
Prosthetic-Fixed	1.15	0.97	1.35	0.103
	**RR**	**95% CI**		**p-val**
**Intensity of Service (given use of a specific service)**				
Diagnostic	0.94	0.91	0.97	< 0.001
Prophylaxis	0.95	0.93	0.98	0.002
Periodontal Maintenance	0.96	0.91	1.01	0.111
Overall Preventive Care^^^	0.96	0.94	0.99	0.016
Periodontal-Non Surgical	1.02	0.96	1.09	0.504
Periodontal-Surgical	1.00	1.00	1.00	0.871
Filling	1.01	0.96	1.07	0.745
Crown	0.96	0.90	1.03	0.247
Extraction*	1.06	0.93	1.20	0.389
Prosthetic-Removable	1.00	0.99	1.01	0.476
Prosthetic-Fixed	1.00	0.999	1.00	0.417

*Complete convergence was not achieved. Estimates based on last concave results.

^Prophylaxis Or Periodontal Maintenance.

## Discussion

We have proposed an innovative approach for assessing dental utilization. The proposed hurdle model allowed us to characterize differences between adults with and without diabetes on three dimensions of dental utilization: the use of dental services, the type of dental care used, and the intensity of each type of service. This model allowed us to decompose dental utilization into multiple components and to examine the impact of diabetes on each of these components separately. This decomposition allowed us to determine whether diabetes impacts the use of services by increasing or decreasing the probability of using any dental services or whether diabetes differentially impacts specific classes of treatment or the frequency with which specific classes of treatment are used. From the perspective of planning the availability of dental services, this is a useful approach because it gives us the ability to identify whether utilization is impacted at the person level or whether the intensity of use of certain procedures is affected.

It is interesting to note that the prevalence of diabetes mellitus in this population was 8.24%, which closely approximates the CDC-stated prevalence in the United States. Our study indicated that individuals with diabetes were less likely to visit a dentist than similar individuals without diabetes. This finding emphasizes the need to increase awareness and support for oral health care among diabetes patients. A similar pattern was reported by Tomar et al. 2000 [31], in a study on dentate adults that concluded that adults with diabetes were less likely than those without to have seen a dentist (65.8% vs. 73.1%) within the 12 month study period. Over the 5-year study period, our results showed a more minor, though statistically significant, difference (84% vs. 88%).

Our results suggest that adults with diabetes receiving dental care had an increased risk of oral complications as evidenced by substantial differences in the mix of services between the two groups. They were more likely to receive periodontal care – maintenance and non-surgical procedures, tooth extractions, and tooth replacing procedures like removable prosthetics. This is not surprising given the established link between diabetes and periodontitis and between periodontitis and tooth-loss.

Taylor et al. 2008 [32], in its review of adverse effects of diabetes on periodontal health, identified 13/17 observational studies that provided consistent evidence of greater prevalence, severity, extent, or progression of at least one manifestation of periodontal disease. Additionally, evidence from three observational studies supported periodontal disease increasing the risk for diabetes complications and no published reports refuted the findings. Kapp et al. 2007 [33], in a survey-based one-year study on a national sample of dentate adults, found that prevalence of tooth-loss among adults with diabetes was 38% and that adults with diabetes had 1.5 times greater odds of having at least one tooth removed compared to non-diabetics. These national level estimates of tooth loss within a single year are higher than our estimates of tooth-loss prevalence (33%) and odds ratio (1.4) in a five year period in the state of Washington. In addition to differences in the study populations, the survey based design of the earlier study may also contribute to differences between the results of the two studies. Studies from Bacic et al [34,35] showed the number of extracted teeth to be significantly greater in the diabetic group than in the control group (14.1 vs. 10.4). Other studies have postulated tooth loss as an inevitable result of periodontal disease that is difficult to control [36-39]. Given that we measured incidence of extractions during the study period and we did not have a diagnosis measure of baseline edentulism among the study participants, we do not have a direct means of comparing our estimates with that of the earlier study. Most of the difference in our tooth loss estimates (measured by number of extractions) between the two groups came from more diabetes patients receiving an extraction rather than more extractions being performed per patient. Furthermore, our findings of higher odds of prosthetics and lower odds of restorations among patients with diabetes are consistent with their increased tooth loss [40-43].

Upon evaluating preventive oral health services, we found that among patients using dental services, those with diabetes were less likely than matched controls to receive overall preventive dental cleanings that included both prophylactic and periodontal maintenance services. When viewed individually, patients with diabetes were less likely to receive prophylaxes but more likely to receive periodontal maintenance codes that unlike the former represent procedures used in the treatment of active oral disease or its maintenance once stabilized [44]. In addition, they received significantly fewer diagnostic procedures. Given previous findings that adults with diabetes had significant oral complications and that regular care provides opportunities for identification, prevention and treatment of periodontal disease, this finding suggests a significant prevention gap and emphasizes the episodic nature of use of dental care in this group.

Some of the statistically significant differences in Table 2 are quite small. The statistical significance of these results may be attributable to our large sample size. Given the substantial and increasing prevalence of diabetes in the population (8.24% in our cohort), even small significant differences in highly used procedures like diagnostic services and preventive care or in less utilized but more expensive procedures like fillings, extractions or removable prosthetics are likely to have meaningful effect on population oral health and health care cost and utilization.

Our study has several additional strengths. First, we have used a propensity score matching method which provides more flexible and robust adjustment for confounding than regression methods provide when the distribution of potential confounding variables differs substantially between comparison groups. As anticipated, adults with diabetes differed significantly from control patients at baseline. After matching procedures were completed, the two groups were found to be similar in the distribution of baseline characteristics. Second, unlike the traditional regression approach, propensity score matching allowed assessment of the sensitivity of results to possible hidden bias. Finally, the use of administrative data rather than self-reported oral care utilization eliminates the potential for recall bias present in survey-based studies.

The methodology used in this study has some limitations. First, the proposed hurdle model assumes independence between the use and intensity (counts) of dental services. This assumption may not be realistic. However, dependence between use and intensity that is attributable to covariates included in our matching procedure will be eliminated via matching. Second, the standard errors of post-matching estimates did not fully account for the estimation of propensity score matching weights, which were assumed fixed and known in these analyses. Replication methods (e.g. bootstrap) could be used to obtain standard error estimates that account for variability in the estimated propensity scores. However, bootstrapped standard errors are very unlikely to alter our results as the study employed a large sample size resulting in highly precise effect estimates. Because separate hypothesis tests were conducted for each class of dental utilization, significance tests should be considered with reference to the number of hypothesis tests that were conducted. Third, because our cohort consisted of patients with well-established diabetes, it is possible that these patients had severe periodontal disease or edentulism at baseline contributing to adverse oral outcomes or fewer dental visits during the study period. The claims data used contained no diagnosis data that detailed periodontal disease severity or the number of teeth at the baseline. However, the results from sensitivity analyses suggest that our inference for most outcomes is robust unless the effects of unobserved confounders such as baseline edentulism were high enough to approximately double the odds of diagnosis of diabetes in our study population. For a few outcomes such as restorations, our results were sensitive to moderate effects of unobserved confounders. The possibility of bias due to unobserved confounding is a limitation of observational studies.

The results of this study show that among adults seeking dental care, those with diabetes have higher utilization of dental surgeries and extractions and therefore may potentially benefit from increased prophylactic services. In future research, this could be directly tested via a randomized trial. Additionally, we plan to study differences in cost patterns associated with classes of dental services to gain insight into the economic consequences of the impact of diabetes on dental services utilization observed in this study.

## Conclusions

Our results suggest the need to encourage appropriate changes in the use of dental care that will help adults with diabetes receive early intervention, possibly reducing subsequent severe oral disease and costs. We recommend that organizations focused on oral health and diabetes care work jointly to initiate programs that educate patients with diabetes about preventive care and promote periodic dental visits. We also recommend that dental carriers not only increase dental coverage for adults with diabetes but also develop incentive products to promote use of preventive and restorative care.

## Competing interests

RJR is an employee and shareholder of Group Health Permanente, the medical group affiliated with Group Health Cooperative. None of the authors have other conflicts of interest to report, and no portion of this work is currently under consideration for publication elsewhere.

## Authors’ contributions

All authors contributed towards the conception and design of the study and critical revision for important intellectual content. MC, RJR and KMN participated in acquisition of data; MC, RH, RI and WEB participated in the analysis and interpretation of data; MC drafted the article. RJR and RI obtained the funding for the study. All authors read and approved the final manuscript.

## Pre-publication history

The pre-publication history for this paper can be accessed here:

http://www.biomedcentral.com/1472-6831/12/20/prepub

## Supplementary Material

Additional file 1 **Appendices [**[21,27]**].** Click here for file
