# Supplementary material for: Evaluating components of dental care utilization among adults with diabetes and matched controls via hurdle models
Source: BMC Oral Health. 2012 Jul 9;12:20. doi: 10.1186/1472-6831-12-20 (PMC3528407; doi:10.1186/1472-6831-12-20)
Supplement: Additional file 1 — Appendices [[21,27]]. [file 1472-6831-12-20-S1.doc]

**Appendix A: Average Exposure Effect**

Matching weights were used to assess differences in outcomes between the two groups through Average Exposure Effects (AEE), defined as a weighted sum:

$$\begin{matrix} \begin{matrix} \end{matrix}(AEE\begin{matrix} ) & \begin{matrix} = & \frac{1}{N^{D}}\sum_{i\in T} \left[\begin{matrix} Y_{i}^{T} & - & \sum_{j\in C_{i}} w_{ij}Y_{j}^{C} \end{matrix}\right] \end{matrix} \end{matrix} \\ \begin{matrix} & & & = & \frac{1}{N^{D}}\begin{matrix} \sum_{i\in T} Y_{i}^{T} & \begin{matrix} - & \frac{1}{N^{D}} \end{matrix} \end{matrix}\sum_{j\in C} w_{j}Y_{j}^{C} \end{matrix} \\ \begin{matrix} & & & = & \frac{1}{N^{D}}\left[\sum_{i\in\Omega^{T}} Y_{i}^{T}-\sum_{j\in\Omega^{C}} w_{j}Y_{j}^{C}\right] \end{matrix}+\frac{1}{N^{D}}\left[\sum_{i\in\Theta^{T}} Y_{i}^{T}-\sum_{j\in\Theta^{C}} w_{j}Y_{j}^{C}\right] \end{matrix}$$

where *N^D^ =* number of diabetic individuals on support in the sample, *Ω* = the entire cohort with common support, Θ = common support for participants that had some dental care. The kernel weight $w_{ij}$ given to the *j^th^* member of the control group when compared with the *i^th^* diabetic adult is defined as where *N_(i)_* is the number of controls matched to the i^th^ diabetic adult and *h_n_* is the chosen bandwidth. Variances of the above AEE estimators were derived using the formula:

$$Var(AEE\begin{matrix} ) & =\begin{matrix} & \frac{1}{(N^{T})^{2}} \end{matrix} \end{matrix}\begin{matrix} \left[\sum_{i\in T} \begin{matrix} Var(Y_{i}^{T}) & + & \sum_{j\in C_{i}} (w_{j})^{2}Var(Y_{j}^{C}) \end{matrix}\right] & \end{matrix}$$

This assumed weights to be fixed and the outcomes to be independent across units. *T* tests with an alpha level of 0.05 were used to detect significant differences between groups.

**Appendix B: Sensitivity Analyses for Unobserved Confounding**

Despite our efforts to control for observable sources of bias via matching, diabetes and dental utilization may be associated because of failure to match on a relevant but unobserved covariate. We conducted sensitivity analyses to examine the degree to which unobserved confounding factors could alter study results. Since measuring selection bias with non-experimental data is not possible, we addressed this problem with the bounding approach proposed by Rosenbaum (2002) [21]. Rosenbaum’s bounds are relatively free of parametric assumptions and provide a single, easily interpretable measure of the degree of confounding necessary to change our inference. This test assumes that an unobserved covariate exists, that affects diabetes status as well as a binary outcome. We further assume that matched participants may differ in their odds of having diabetes by a factor of as a result of differences in this unobserved covariate (for example, tooth-loss at baseline). The larger the value of for which the inference on the effect of diabetes on dental outcomes is unaffected, the less likely is the explanation that the detected effect is due to hidden bias.

If the probability of subject *j* to have diagnosed with diabetes is$\omega_{j}$, the odds are then $\omega_{j}$/(1-$\omega_{j}$). The log odds can be modeled as a generalized function of a vector of observed characteristics $x_{j}$and an unobserved binary covariate$u_{j}\in\left\{0,1\right\}$, such that$\log(\omega_{j}/(1-\omega_{j}))=\kappa x_{j}+\gamma u_{j}$, where $\gamma$ is the effect of $u_{j}$ on the log odds of diabetes [21]. Given a matched pair of individuals *i* and *j*, both having identical observed covariates $x_{i}$=$x_{j}$, the odds ratio is given by OR =$\exp\left\{\begin{matrix} \end{matrix}\gamma\left(u_{i}-u_{j}\right)\begin{matrix} \end{matrix}\right\}$. So, if there are either no differences in unobserved variables ($u_{i}=u_{j}$) or if unobserved variables have no influence on the probability of diabetes (*γ* = 0), the odds ratio is 1 corresponding to equal probability of diabetes. Rosenbaum [21] shows that the OR is bounded as: $(1/e^{\gamma})\leq OR\leq e^{\gamma}$. Defining$\Gamma=e^{\gamma}$, setting $\gamma$ = 0 and $\Gamma$= 1 implies that no hidden bias exists, and hence is equivalent to the conditional independence assumption underlying the matching method analysis. Both matched individuals have the same probability of diabetes only if $\Gamma$ = 1. For $\Gamma$ = 2, individuals who appear to be similar (in terms of *x*) could differ in their odds of having diabetes by as much as a factor of 2. In this sense, $\Gamma$ is a measure of the degree of departure from a study that is free of hidden bias [21].

To determine the strength of hidden confounding necessary to undermine our conclusions based on the observed association between diabetes and the outcome, we used the Mhbounds [27] (version 1.1.5) implementation of Rosenbaum’s method for each binary outcome where significant differences between patients with and without diabetes were found (Table B.1). For each outcome, we report the minimum value for that would be required to change our inference about the association between diabetes and dental outcomes. High values of indicate that we would need to postulate extremely strong hidden bias in order to explain the observed association between diabetes and dental outcomes, while smaller values of suggest that modest confounding by unobserved covariates could potentially explain the observed result.

The lack of statistical significance observed for high values of (> 1.75) for probability of dental visit and, given a dental visit, for probability of overall preventive care, extractions and removable prosthetics, suggests robustness of the estimates to hidden bias. However, for a few classes (e.g. restorations), estimates were more sensitive. Regression adjustment will eliminate some of the bias originating from imbalances between the two groups due to conditioning on dental visit. Associations between diabetes and these outcomes should be interpreted more cautiously.

| **Table B.1. Sensitivity of Estimates for Use and Type of Dental Services following Propensity Score Matching (bw=0.008).** | |
| --- | --- |
| **Service Use** | Level of Gamma |
| Dental Visit | 2.00 |
| **Services by Class Given Dental Visit** | |
| Prophylaxis | 1.75 |
| Periodontal Maintenance | 1.25 |
| Periodontal-Non Surgical | 1.25 |
| Overall Preventive Care | 2.00 |
| Fillings | 1.25 |
| Crowns | 1.25 |
| Extractions | 2.00 |
| Prosthetics Removable | 2.00 |

**References**

1. Rosenbaum PR. Observational Studies. *2nd ed. New York, NY: Springer 2002*.
2. Becker SO & Caliendo M. Mhbounds - Sensitivity Analysis for Average Treatment Effects. [*IZA Discussion Papers*](http://ideas.repec.org/s/iza/izadps.html) 2007; 2542
3. Mantel, N. and Haenszel, W. (1959) Statistical aspects of the analysis of data from retrospective studies of disease. J Natl Cancer Inst 22, 719-748.

**Appendix C: Sensitivity to Chosen Bandwidth**

Choosing a bandwidth is analogous to the problem of choosing the number of neighbors in a nearest neighbor setting. The bandwidth choice is therefore a compromise between a small variance and an unbiased estimate of the true density function. Another value, h_n_ = 0.03, was used to test the sensitivity of our estimates to chosen bandwidths, Table C.1 & Table C.2.

| **Table C.1. Post Matching AEE Estimates (bw = 0.03) for Effect of Diabetes on Dental Outcomes** | | | | | | | | | | |
| --- | --- | --- | --- | --- | --- | --- | --- | --- | --- | --- |
|  | **Probability of Utilization** | | | | |  | **Average Number of Procedures^↑^** | | | |
|  | **Treated** | | **Controls** | **Difference** | **pvalue** |  | **Treated** | **Controls** | **Difference** | **pvalue** |
| ***Dental Services*** | 0.84 | | 0.87 | -0.03 | < 0.001 |  | 22.61 | 24.56 | -1.95 | < 0.001 |
| **Services by Dental Class given at least one Dental Visit** | | | | | |  | | | | |
| Diagnostic | | 0.98 | 0.98 | 0.00 | 0.283 |  | 12.03 | 12.89 | -0.86 | < 0.001 |
| Prophylaxis | | 0.72 | 0.77 | -0.05 | < 0.001 |  | 4.12 | 4.75 | -0.63 | < 0.001 |
| Periodontal Maintenance | | 0.29 | 0.25 | 0.04 | < 0.001 |  | 1.94 | 1.79 | 0.16 | 0.07 |
| Periodontal-Non Surgical | | 0.25 | 0.20 | 0.05 | < 0.001 |  | 0.90 | 0.71 | 0.19 | < 0.001 |
| Periodontal-Surgical | | 0.03 | 0.03 | 0.00 | 0.182 |  | 0.04 | 0.05 | -0.01 | 0.10 |
| Overall Preventive Care**^** | | 0.86 | 0.89 | -0.03 | < 0.001 |  | 6.06 | 6.54 | -0.48 | < 0.001 |
| Fillings | | 0.67 | 0.72 | -0.05 | < 0.001 |  | 2.79 | 2.98 | -0.19 | 0.02 |
| Crowns | | 0.48 | 0.53 | -0.05 | < 0.001 |  | 1.04 | 1.18 | -0.14 | 0.001 |
| Extractions | | 0.33 | 0.27 | 0.06 | < 0.001 |  | 0.95 | 0.69 | 0.26 | < 0.001 |
| Prosthetic -Fixed | | 0.11 | 0.10 | 0.01 | 0.048 |  | 0.41 | 0.39 | 0.02 | 0.30 |
| Prosthetic-Removable | | 0.17 | 0.13 | 0.04 | < 0.001 |  | 0.39 | 0.29 | 0.09 | < 0.001 |
| *Overall | |  |  |  |  |  | 26.88 | 28.10 | -1.22 | 0.00 |
| ***^↑^ estimates conditional on at least one dental visit but unconditional on at least one service in a specific class. The denominator includes those with no utilization for a given service***  ******* ***includes any dental service, not restricted to those mentioned***  ***^ Prophylaxis or Periodontal Maintenance*** | | | | | | | | | | |

| **Table C.2. Post-Matching Diabetes Effect estimates (bw=0.03) from Hurdle Model** | | | | | |
| --- | --- | --- | --- | --- | --- |
|  |  | **OR** | **95% CI** | | **p-val** |
| **Dental Visit** |  | 0.75 | 0.64 | 0.87 | < 0.001 |
|  | | | | | |
| **Use of Service given at least one Dental Visit** | | | | | |
| Diagnostic |  | 0.93 | 0.62 | 1.38 | 0.709 |
| Prophylaxis |  | 0.76 | 0.67 | 0.86 | < 0.001 |
| Periodontal Maintenance |  | 1.22 | 1.08 | 1.37 | 0.001 |
| Overall Preventive Care**^** |  | 0.76 | 0.64 | 0.89 | 0.001 |
| Periodontal-Non Surgical |  | 1.30 | 1.15 | 1.47 | < 0.001 |
| Periodontal-Surgical |  | 0.86 | 0.62 | 1.18 | 0.335 |
| Filling |  | 0.79 | 0.70 | 0.88 | < 0.001 |
| Crown |  | 0.82 | 0.74 | 0.91 | < 0.001 |
| Extraction |  | 1.36 | 1.21 | 1.52 | < 0.001 |
| Prosthetic-Removable |  | 1.36 | 1.17 | 1.57 | < 0.001 |
| Prosthetic-Fixed |  | 1.16 | 0.99 | 1.36 | 0.075 |
| **Intensity of Service (given use of a specific service)** |  | **RR** | **95% CI** | | **p-val** |
| Diagnostic |  | 0.94 | 0.91 | 0.97 | < 0.001 |
| Prophylaxis |  | 0.93 | 0.90 | 0.97 | < 0.001 |
| Periodontal Maintenance |  | 0.95 | 0.88 | 1.02 | 0.137 |
| Overall Preventive Care**^** |  | 0.96 | 0.93 | 0.99 | 0.005 |
| Periodontal-Non Surgical |  | 1.02 | 0.96 | 1.08 | 0.553 |
| Periodontal-Surgical |  | 0.94 | 0.79 | 1.11 | 0.480 |
| Filling |  | 1.00 | 0.98 | 1.03 | 0.797 |
| Crown |  | 0.98 | 0.92 | 1.03 | 0.374 |
| Extraction |  | - | - | - | - |
| Prosthetic-Removable |  | 1.06 | 0.89 | 1.26 | 0.526 |
| Prosthetic-Fixed |  | 0.96 | 0.88 | 1.04 | 0.318 |

- *Model did not converge*

*^ Prophylaxis or Periodontal Maintenance*

**Appendix D: Procedure classifications based on American Dental Association Coding system - Current Dental Terminology (CDT)**

| **CDT Class** | **CDT Code Range** | **CDT Codes** |
| --- | --- | --- |
| ***Diagnostic*** | 0110 -0 999 | 110,120,130,140,150,160,170,180,210,220,230,240,250,260,270,272,273,  274,277,290,310,320,321,322,330,340,350,415,420,425,431,460,470,471,  472,473,474,475,476,480,482,484,501,502,990,999 |
| ****Periodontal*** | 4210 - 4999 |  |
| Maintenance |  | 4910 |
| Non Surgical |  | 4341,4342,4355,4381 |
| Surgical |  | 4240,4241,4245,4260,4261,4263,4264,4265,4266,4267 |
| ***Preventive*** | 1110 - 1999 |  |
| Prophylaxis |  | 1110,1120 |
| Fluoride |  | 1203,1204,1206 |
| Others |  | 1130,1201,1205,1207,1221,1231,1310,1320,1330,1351,1510,1520,1525,  1550,1990,1999 |
| ***Restorative*** | 2110 - 2999 |  |
| Crowns |  | 2710,2720,2721,2722,2740,2750,2751,2752,2780,2781,2782,2783,2790,  2791,2792,2794,2799 |
| Fillings |  | 2140,2150,2160,2161,2330,2331,2332,2335,2337,2340,2380,2381,2382,  2385,2386,2387,2388,2390,2391,2392,2393,2394 |
| ***Oral Surgery*** | 7110 - 7999 |  |
| Extractions |  | 7110,7111,7120,7130,7140,7210,7220,7230,7240,7241,7250 |
| ***Prosthodontic*** |  |  |
| Fixed | 6010 - 6999 | 6010,6020,6040,6050,6053,6054,6055,6056,6057,6058,6059,6060,6061,  6062,6064,6065,6066,60676068,6069,6071,6072,6073,6076,6077,6078,  6079,6080,6090,6095,6100,6190,6199,6205,6210,6211,6212,6240,6241, |
|  |  | 6242,6245,6250,6251,6252,6253,6545,6548,6600,6602,6607,6609,6611,  6615,66306640,6710,6720,6721,6740,6750,6751,6752,6780,6781,6782,  6783,6790,6791,6792,6793,6920,6930, 6940,6950,6960,6970,6971,6972, |
|  |  | 6973,6975,6976,6980,6990,6999 |
| Removable | 5110 - 5999 | 5110,5120,5130,5140,5211,5212,5213,5214,5215,5218,5225,5226,5241,  5281,5310,5320,5410,5411,5421,5422,5510,5520,5610,5620,5630,5640,  5650,5660,5670,5671,5710,5711,5720,5721,5730,5731,5740,5741,5750, |
|  |  | 5751,5760,5761,5810,5811,5820,5821,5822,5823,5850,5851,5860,5861,  5862,5867,5875,5899,5922,5931,5932,5933,5953,5954,5982,5986,5987,  5988,5999 |
| ***CDT: Current Dental Terminology***  ** In an earlier validation study^↑^, we determined that the sensitivity, specificity and positive predictive value of our periodontal class codes were 84%, 44% and 80% respectively in identifying persons with moderate to severe periodontitis. In a sub analysis, however, the three measures were 97%, 96%, 84% respectively in identifying persons with either maintained or moderate to severe periodontitis.*  *^↑^ Spangler L et al. 2010. “Administrative Data for Epidemiologic Research: Case Study to Identify Persons with Periodontitis.” Periodontology 2000. [In Press 2011]* | | |
